# Molecular surveillance and antimicrobial susceptibility profile of bacterial contamination in pastries of Iranian confectioneries: a public health concern

**DOI:** 10.3389/fmicb.2024.1494623

**Published:** 2024-12-04

**Authors:** Shiva Hosseini, Tahereh Motallebirad, Mohammad Reza Mohammadi, Mehdi Safarabadi, Zeynab Beheshti, Mohammad Ali Orouji, Omid Mardanshah, Davood Azadi

**Affiliations:** 1 Department of Genetics, Marvdasht Branch, Islamic Azad University, Marvdasht, Iran; 2 Department of Research and Development, Satras Biotechnology Company, Islamic Azad University of Khomein, Khomein, Iran; 3 Department of Bacteriology, Faculty of Medical Sciences, Tarbiat Modares University, Tehran, Iran; 4 Department of Nursing, Khomein University of Medical Sciences, Khomein, Iran; 5 Department of Nursing, Khomein Branch, Islamic Azad University, Khomein, Iran; 6 Department of Parasitology and Mycology, School of Medicine, Isfahan University of Medical Sciences, Isfahan, Iran; 7 Department of Biology, Faculty of Basic Sciences, Lorestan University, Khorramabad, Iran

**Keywords:** pastries, microbial pollution, AST, 16s rRNA, microbial contamination

## Abstract

**Introduction:**

Microbial contamination in food products such as pastries, poses a significant public health concern due to the potential risks of foodborne infection and outbreak, Therefore, to prevent these infections, it is essential to investigate the frequency and extent of microbial contamination as well as the level of drug resistance in pastries. Due to this issue, our study aimed to assess the microbial diversity and the drug susceptibility patterns of microbial pollutants in pastry shops in Markazi province, Iran.

**Methods:**

The study involved collecting 120 pastry samples from 30 pastry shops in Markazi province, Iran. The isolates were identified using a series of biochemical, phenotypic, and molecular assays, including specific PCR and 16S rRNA gene sequencing. Drug susceptibility testing (AST) was performed by using the Kirby-Bauer method according to the CLSI 2023 guidelines.

**Results and discussion:**

A total of 56 isolates (46.66%) were recovered from 120 pastries samples, The most prevalent species isolated in the current study were *S. aureus* 12 isolates (21.43%), *M. luteus* 7 isolates (12.5%), *E. coli* 7 isolates (12.5%), *S. warneri* 6 isolates (11.12%), 6 isolates of *S. succinus* (11.12%), *B. cereus* 5 isolates (10.7%), Nocardia 4 isolates (7.15%), K. pneumoniae 3 isolates (5.35%), S. epidermidis 3 isolates (5.35%), and *E. faecium* 3 isolates (5.35%). The isolates showed the most sensitivity to imipenem and trimethoprim-sulfamethoxazole and the least sensitivity to erythromycin and tetracycline. The AST showed that 7 isolates of *S. aureus* were MRSA, 3 isolates of *E. coli* and, 2 isolates of *K. pneumoniae* were identified as ESBL. In conclusion, the results of the current study showed that the microbial contamination of pastries produced in confectionaries of Markazi province was not in standard ranges. These problems may be related to fecal contamination of pastries or lack of hygiene by handlers and it is urgent to develop the standards of hygiene of food handling techniques and aseptic pastry production in confectioneries.

## 1 Introduction

In recent years, there has been an increasing concern over the safety and quality of food products, including pastries. This concern arises from the potential risks associated with microbial contamination, which can lead to foodborne infection and outbreaks. Pastries are popular food items consumed worldwide, appreciated for their taste and variety. However, microbial contamination in pastries has become a significant concern due to the potential transmission of infectious microorganisms. These microbial infections can pose a risk to public health and lead to foodborne illnesses. Microbial contaminants in pastries can include pathogenic bacteria, such as Salmonella, *E. coli*, and *S. aureus*, as well as spoilage bacteria, yeasts, and molds. Therefore, understanding the types of microorganisms present in pastries, the routes of pastry contamination, and the importance of detecting microbial contamination in pastries is crucial for ensuring food safety (Motallebirad et al., 2023).

The presence of these microorganisms in pastries can be attributed to various factors. Firstly, the ingredients used in pastry production may already be contaminated before they are incorporated into the final product. Raw materials such as flour, eggs, milk, and butter can harbor microorganisms, and if not properly handled and stored, can contaminate the pastry dough. Additionally, poor hygiene practices during the preparation process, such as inadequate hand washing or improper cleaning of equipment and utensils, can introduce microbial contaminants. Moreover, the environment in which pastries are produced and stored can also contribute to microbial contamination. Contaminated surfaces, air quality, and pests can all introduce microorganisms into the pastry production area. Improper storage conditions, such as inadequate temperature control or extended shelf life, can allow microbial growth and increase the risk of contamination (Velasquez-Orta et al., 2011; Kopittke et al., 2019).

Detecting and identifying microbial contaminants is crucial for preventing foodborne illnesses and outbreaks. Rapid and accurate detection methods are essential for ensuring the safety of pastries consumed by the public. Phenotypic methods, such as microbial culturing and biochemical tests, can provide initial identification of the microorganisms present. However, molecular methods, including polymerase chain reaction (PCR) and DNA sequencing, offer higher specificity and sensitivity in detecting and identifying microbial contaminants, enabling a more comprehensive analysis of the microbial community in pastries (Vidyadharani et al., 2022; Aladhadh, 2023).

Determining antimicrobial resistance in bacteria isolated is crucial. The pastries’ microbial pollution has been associated with foodborne illnesses caused by resistant pathogens such as MRSA and ESBL-positive Enterobacteriaceae. Thereby, determining antimicrobial resistance, including MRSA and ESBL, helps assess the potential risks associated with these food items and guides the development of effective preventive measures. Due to this issue understanding the resistance patterns of microbial pollution of pastries, can implement targeted interventions to mitigate the spread of resistant strains and safeguard public health (Okaiyeto et al., 2024).

Food safety especially pastries, is an essential public health issue, particularly in regions where foodborne diseases remain a significant health burden (Madilo et al., 2024). Markazi province, located in central Iran, is an area with diverse climatic conditions ranging from cold winters to warm, dry summers. This climate can influence the handling and storage conditions of food, particularly pastries, which are sensitive to environmental changes that may promote microbial growth if not managed properly. Markazi’s population includes urban and rural communities with varying access to essential amenities. While most urban areas benefit from relatively stable water supplies and access to sanitary facilities, rural areas may experience challenges in water quality and sanitation. This disparity can impact hygiene practices and increase the risk of food contamination in smaller, rural pastry shops. The area’s literacy rate is high, yet food handler training on safety and hygiene in local confectioneries varies significantly, often depending on regulatory oversight and the resources of individual shops. In this context, the pastry industry plays an important role, with licensing and training regulations managed by local health authorities (Goodarzi et al., 2023). However, adherence to these regulations can vary, affecting food safety standards. Additionally, antibiotic use in the region is also noteworthy, as self-medication and over-the-counter antibiotic purchases are common. This pattern of antibiotic consumption could contribute to the emergence of drug-resistant bacteria in food products, posing additional health risks. Among children, who are particularly vulnerable to foodborne illnesses, such incidents can lead to severe health outcomes, stressing the importance of stringent hygiene in food preparation (Nazari et al., 2023).

Pastries, a popular snack in Markazi, are made with ingredients like flour, eggs, and dairy, which, if not stored or handled correctly, can harbor pathogens. The frequent use of creamy fillings and toppings further increases the risk of microbial contamination. Thus, ensuring hygiene in pastry production is crucial to prevent outbreaks of foodborne diseases. Therefore, due to provide valuable insights into the prevalence, diversity, and potential health risks associated with microbial contamination in pastries, and also, development of effective preventive strategies, quality control measures, and regulatory guidelines to ensure the safety and hygiene of pastries in the food industry, conducting a study on microbial contamination in pastries holds great importance for health facility organization. Due to these issues in this study, we aim to explore the species diversity of microbial contamination and AST of different confections in the pastries shop of the Markazi province of Iran. By investigating microbial contamination in pastries, we strive to contribute to the scientific understanding and promotion of food safety practices in the pastry industry.

## 2 Materials and methods

### 2.1 Sampling, isolation, and conventional identification

In a descriptive study, conducted from September to December 2023, a total of 120 pastry samples including sweet pastries, cakes, and similar baked goods were collected from 30 pastry shops across various cities in Markazi province, Iran. The samples were processed within 12 h of collection in the microbiology laboratory of Khomein University of Medical Sciences. To isolate the bacteria, 10 grams of the collected samples were suspended in 90 mL sterile phosphate buffered saline, pH 7.4 (PBS) for 10 min. 10 mL of the suspension was added to 10 mL of nutrient broth (HiMedia, India). The tubes were incubated at 37°C for 18–24 h. Then, 10 μL of the enriched cultures were inoculated in blood agar with 5% sheep blood, thioglycolate, MacConkey agar, and sothon agar and incubated at 37°C for 48 h. A pure culture was then prepared from all the colonies obtained from the samples to perform diagnostic tests. For initial identification of bacteria, the following tests were conducted: pigment production, growth rate, nitrate reduction, urea, casein, starch, and growth in 0.4% gelatin, catalase, oxidase, TSI, IMVIC, MSA, hemolysis, and CAMP test. Further identification was carried out using molecular tests (Siavashifar et al., 2021; Motallebirad et al., 2023).

### 2.2 Molecular identification

#### 2.2.1 DNA extraction

Chromosomal DNA was extracted using a simple boiling method (Rahdar et al., 2017). In brief, a few colonies of each isolate were added to 100 μL of TE buffer (10 mM Tris, 1 mM EDTA, pH 7.8) and boiled for 10 min at 100°C. After centrifugation of bacterial suspensions at 9,000 × g for 30 s at 4°C, Precipitated DNA was resuspended in 50 μL of Milli-Q water and stored at −20°C.

#### 2.2.2 Species identification

For species identification, PCR amplification and sequence analysis of the 16S rRNA gene were used, as described by Azadi et al. (2020). The sequencing was done by Pishgam Biotech Company (Iran). The sequences were aligned by the Clustal W v2.0 software with the existing sequences of our isolates and compared with sequences of closely related bacterial species obtained from the GenBank database using the jPhydit program version 1.1.3 (Jeon et al., 2005).

### 2.3 Antibiotic susceptibility testing

The AST for all isolates was performed using the Kirby- Bauer method on Mueller Hinton agar (MHA), following the CLSI-2021 criteria. MHA with 5% sheep blood was used for fastidious strains such as streptococci (Clinical and Laboratory Standards Institute, 2021). The antibiotic disks used in this study were: Trimethoprim/Sulfamethoxazole (25 μg), Erythromycin (15 μg), levofloxacin (5 μg), Vancomycin (30 μg), Clindamycin (2 μg), Penicillin (10 μg), Amoxicillin-clavulanic acid (30 μg), Cefoxitin cefepime (30 μg), ceftazidim, Cefotaxime (30 μg), Ceftriaxone (30 μg), Imipenem, Tetracycline (30 μg), and Clarithromycin (15 μg) (Mast, Merseyside, United Kingdom). The isolates were cultured with a sterile cotton swab on an eight-cm plate containing MHA medium. Then, antibiotic disks were positioned on the surface of the culture medium, at a standardized distance of 2.5 cm from one another. After 24 h, the diameter of the zone of inhibition was measured and analyzed. The breakpoints for resistance and susceptibility were defined based on CLSI recommendations. Quality control of minimum inhibitory concentrations (MICs) was carried out by testing CLSI-recommended reference strains, including *E. faecalis* ATCC 29212 and *S. aureus* ATCC 29213. Multidrug resistance (MDR) was described as resistance to at least three different classes of antibiotics that were tested in this study.

#### 2.3.1 Determination of MRSA and ESBLs resistant isolates

Methicillin-resistant *S. aureus* (MRSA) isolates were characterized using an oxacillin agar screening test, which involved the use of MHA containing 4% NaCl and 2 μg/mL oxacillin. The growth of bacteria in the presence of oxacillin was considered a positive indication of MRSA strain (Ahmadi et al., 2019). Two standard strains, including MSSA ATCC 29213 and MRSA ATCC 43300, were applied as negative and positive controls, respectively. The identification of extended-spectrum beta-lactamases (ESBL) producing isolates was conducted using a phenotypic test that followed the CLSI 2023 criteria. In this test, ceftazidim (30 μg) and a combination of ceftazidim with clavulanic acid (ceftazidim + clavulanic acid, 30/10 μg) disks were utilized. If the zone of inhibition around the combination disks was 5 mm greater than that around the ceftazidim disk alone, it was considered a positive indication of ESBL production (Clinical and Laboratory Standards Institute, 2021).

### 2.4 Statistical analysis

Statistical analysis was performed on the collected data to determine the prevalence and distribution of microbial contaminants across different pastry types and to evaluate their antimicrobial resistance profiles. Descriptive statistics, including frequencies and percentages, were used to summarize the prevalence of contamination and antimicrobial resistance for each isolate. Data were analyzed using SPSS version 23.0.1.

## 3 Results

In the present study, 120 samples were collected in the following order from 30 pastry shops in Markazi province. The type of pastries collected include creamy pastry 30 samples (25%), Danish 30 samples (25%), wet cake 20 samples (16.6%), cheesecake 20 samples (16.6%), doughnuts 20 samples (16.6%). The details of samples and isolates are presented in Table 1.

**Table 1:** Sample profile, and biochemical and molecular features of bacterial isolates recovered from pastries in Markazi province of Iran.

	Sample profile	Biochemical features													16S rRNA analysis	
No of isolates	Designation	Type of pastries	Polymicrobial	Gram stain	Catalase	Oxidase	Coagulase	MSA	Bile esculin	IMVIC^*^	Urease	Lysozyme Resistance	Decomposition of Xanthene	Decomposition of Hypoxanthine	Similarity (%)^a^	Identification
2	PA1,PA2	Doughnut/creamy	−	B + ^*^	+	−	−	−	−	−	−	+	+	−	99.82	*N. asiatica*
2	PA3, PA4	Doughnut/ Danish	+	B+	+	−	−	−	−	−	−	+	−	+	100	*N. asteroides*
12	PA5, PA6, PA7, PA8, PA9, PA10, PA11, PA12, PA13, PA14, PA15, PA16	Creamy/cheese cake/wet cake/Danish/Doughnut	+	C+	+	−	+	+	−	−	+	−	−	−	99.8–100	*S. aureus*
7	PA17, PA18, PA19, PA20, PA21, PA22, PA23	Creamy/cheese cake/wet cake	+	B-	+	−	−	−	−	++ − -	−	−	−	−	99.78–100	*E. coli*
3	PA24, PA25, PA26	Creamy/wet cake	+	B-	+	−	−	−	−	- − ++	+	−	−	−	99.9	*K. pneumoniae*
3	PA27, PA28, PA29	Creamy/doughnuts	−	C+	+	−	−	−	−	−	+	−	−	−	100	*S. epidermidis*
3	PA30, PA31, PA32	Creamy/cheese cake	−	C+	−	−	−	−	+	−	−	−	−	−	100	*E. faecium*
7	PA33, PA34, PA35, PA36, PA37, PA38, PA39	Creamy/wet cake/Danish/Doughnut	+	C+	+	+	−	−	−	−	−	−	−	−	9.98–100	*M. luteus*
6	PA40, PA41, PA42, PA43, PA44, PA45,	Creamy/cheese cake/wet cake/Danish/Doughnut	+	C+	+	−	−	−	−	−	+	−	−	−	100	*S. warneri*
6	PA46, PA47, PA48, PA49, PA50, PA51	Creamy/cheese cake/wet cake/	+	C+	+	−	−	−	−	−	+	−	−	−	99.9	*S. succinus*
5	PA52, PA53, PA54, PA55, PA56	Creamy/Danish/Doughnut	+	B+	+	−	−	−	−	- − ++	−	−	−	−	100	*B. cereus*

IMVIC, Indole, Methyl red, Voges-Proskauer, citrate. B+, Bacilli gram positive. C+, Cocci gram positive.

Based on phenotypic and molecular assays that were applied, a total of 56 isolates (46.66%) were recovered from 120 pastry samples. All sampling sites were positive for bacterial pollution. Among all isolates, 20 (35.7.6%) isolates were recovered from creamy pastries, 14 (25%) isolates were recovered from cheesecake, 10 (17.87%) isolates were recovered from the wet cake, 6 (10.71%) isolates were recovered from Danish, and 6 (10.71%) isolates were recovered from doughnuts. A number of 80 (66.66%) samples were contaminated with fungi, gram-positive and gram-negative bacteria. Fungal species were isolated from the rest of the samples or no growth was observed, so they were excluded from the study. The specifications of the samples and the results of molecular and biochemical tests of isolates are given in Table 1.

The analysis of partial 16S rRNA gene sequences of the isolates revealed that specific nucleotide signatures were present for each genus. For gram negative bacteria the signatures were observed at positions 70–98 (U-A), 843 (C), 1,008–1,021 (C-G), 139–224 (G-C), 1,189 (C), 1,308–1,329 (C-G), and1244–129 (C-G), for gram positive bacteria the signature was placed at position positions 70–98 (A-T), 293–304 (G-T), 307 (C), 328 (T), 614–626 (A-T), 631(G), 661–744 (G-C), 825–875 (A-T), 824–876 (T-A), 843 (C), and 1,122–1,151 (A-T) and for *Nocardia* isolates, the signatures were observed at positions 70–98 (A-T), 307 (C), 293–304 (G-T), 614–626 (A-T), 631 (G), 328 (T), 824–876 (T-A), 661–744 (G-C), 825–875 (A-T), 843 (C), and 1,122–1,151 (A-T) (Igolkina et al., 2018). Based on these results, the isolates belonged to 7 genera and 11 validated species. The most prevalent species isolated in the current study were including *S. aureus* 12 isolates (21.43%), *M. luteus* 7 isolates (12.5%), *E. coli* 7 isolates (12.5%), *S. warneri* 6 isolates (11.12%), 6 isolates of *S. succinus* (11.12%), *B. cereus* 5 isolates (10.7%), *Nocardia (N. asiatica and N. asteroides)* 4 isolates (7.15%), *K. pneumoniae* 3 isolates (5.35%), *S. epidermidis* 3 isolates (5.35%), and *E. faecium* 3 isolates (5.35%).

**Figure 1:**
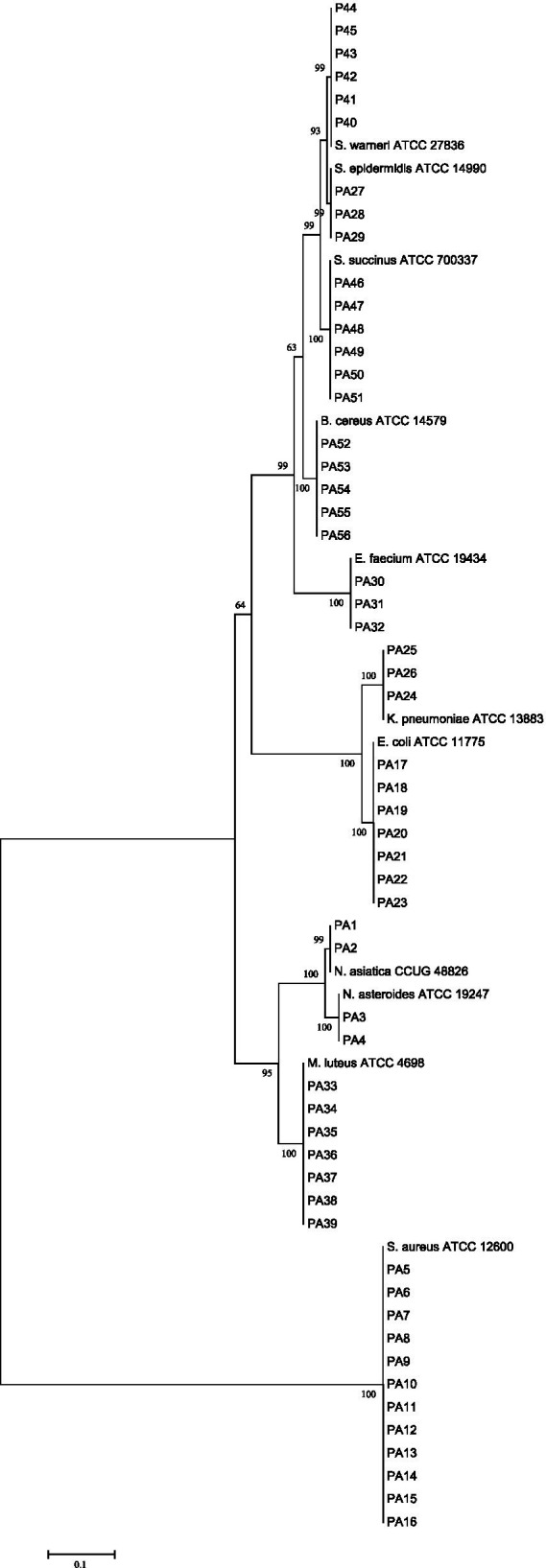
16S rRNA sequence based phylogenetic tree for our clinical isolates and nearest standard species by using the neighbor-joining method depicted by MEG A8 software. At each node bootstrapping values are represented.

The phylogenetic relationship between our isolates and valid established species was depicted in a phylogenetic tree of the 16S rRNA gene, with a high bootstrap value, using the neighbor-joining method. The tree was generated using MEGA 8 software, and it is depicted in Figure 1.

### 3.1 16S rRNA gene sequence accession numbers

The GenBank accession numbers for the 16S rRNA gene sequences of isolated bacteria in this study are listed below, isolate PA1 *N. asiatica* (OP520910), isolate PA3 *N. asteroides* (PV077341), isolate PA17 *E. coli* (PV077340), isolate PA24 *K. pneumoniae* (PV077343), isolate PA5 *S. aureus* (PV077342), isolate PA27 *S. epidermidis* (PV077344), isolate PA40 *S. warneri* (PZ413541), and isolate PA30 *E. faecium* (PV077345).

### 3.2 AST results

The results of AST of all isolates based on CLSI standards showed that the isolates were most susceptible to imipenem and trimethoprim-sulfamethoxazole, and least susceptible to erythromycin, tetracycline, and clarithromycin. Additionally, the AST results for our isolates showed that 7 out of 12 isolates of *S. aureus* were MRSA, 3 out of 7 isolates of *E. coli*, and, 2 isolates of *K. pneumoniae* were identified as ESBL producing strain. Detailed results of the AST are provided in Table 2.

**Table 2:** AST results of clinical isolates examined in the study based on disk diffusion method.

Isolate	Trimethoprim/sulfamethoxazole	Erythromycin	Levofloxacin	Vancomycin	Clindamycin	Penicillin	Amoxicillin- clavulanic acid	Cefepime	Ceftazidim	Cefotaxime	Ceftriaxone	Imipenem	Tetracycline	Clarithromycin	Drug susceptibility pattern
*N. asiatica*	S	S	S	–	S	–	R	S	–	S	S	S	S	R	
*N. asteroides*	S	S	R	–	S	–	R	S	–	S	S	S	S	R	
*S. succinus*	R	S	R	–	S	–	R	S	–	S	S	S	S	S	
*E. coli*	S	R	I	–	R	–	S	R	R	–	R	S	R	–	ESBL
*K. pneumoniae*	S	I	S	–	I	–	S	R	R	–	R	S	S	–	ESBL
*M. luteus*	S	R	S	S	R	R	S	S	S	-	S	S	R	S	
*S. warneri*	S	R	R	–	R	–	R	R	S	-	R	S	S	–	MDR
*S. aureus*	R	R	R	S	R	R	S	–	–	S	S	-	R	R	MRSA
*S. epidermidis*	S	R	R	S	S	S	S	–	–	S	S	-	R	R	MDR
*Enterococcus faecium*	R	R	R	S	S	R	S	R	–	S	S	R	S	S	MDR
*B. cereus*	S	S	R	S	S	S	S	–	–	S	S	S	R	R	

## 4 Discussion

The investigation into microbial contamination within pastry shops holds significant importance in safeguarding public health and ensuring the integrity of food products. With pastries being a staple of many diets, their consumption presents a potential avenue for exposure to harmful microorganisms, thereby elevating the risk of foodborne illnesses and outbreaks (Okpala and Korzeniowska, 2023). Microbial contamination poses a multifaceted challenge, transcending mere food safety concerns to encompass broader public health implications. Inadequate hygiene practices, improper food handling, and environmental factors within pastry production and storage facilities can all contribute to the proliferation of pathogenic microorganisms. These contaminants, ranging from bacteria to fungi, have the potential to cause a spectrum of illnesses, from mild gastrointestinal discomfort to severe infections, particularly in vulnerable populations (Zhou and Ali, 2024). Due to this issue, this study aimed to shed light on the prevalence and potential risks of bacterial species in different pastries and provide efforts to control and prevent infections caused by these microorganisms.

In our study, we collected a total of 120 pastry samples from 30 pastry shops in Markazi province. From there, we were able to recover 56 isolates, of the samples which indicates a significant level of microbial contamination in the pastries is 46.66%. One important finding is that all sampling sites were positive for bacterial pollution. Our result correlated with other studies such as studies conducted by Sharifzadeh et al. (2016) and Hassanzadazar et al. (2018) the prevalence and types of microbial contaminants in pastries were consistent with our findings, such that the prevalence rate was 48.7 to 56.2%, additionally, creamy pastries and wet cake were commonly found to have higher levels of contamination. However, the specific genera and species of microbial contaminants may vary between studies and regions. These results highlight the widespread presence of microbial contaminants in pastry shops and the importance of conducting local studies to understand the specific microbial contamination patterns in a particular area.

Our results showed that creamy pastries exhibited the highest levels of contamination, a finding likely attributed to their specific ingredients, preparation methods, and storage conditions. Cream-based fillings and toppings are more susceptible to microbial growth due to their high moisture content, nutrient availability, and relatively neutral pH, all of which provide an ideal environment for bacterial proliferation. Additionally, the preparation of creamy pastries often involves minimal baking or partial cooking, meaning that microbial contaminants present in raw ingredients may survive the process and persist in the final product. Furthermore, the extended refrigeration required to maintain the texture and taste of these pastries can inadvertently promote the growth of psychrotrophic bacteria, which thrive in cool, moist conditions.

These factors highlight the urgent need for enhanced hygiene standards in confectioneries, particularly in practices related to ingredient handling, food preparation, and storage. Regular cleaning and sanitization of equipment, along with proper refrigeration practices, are essential to minimize contamination risks. Moreover, stricter temperature control protocols during storage and transport are crucial to ensure that these pastries remain safe for consumption. Confectionery staff should also receive targeted training on hygiene practices, focusing on minimizing cross-contamination during the preparation and handling of high-risk items like creamy pastries. Implementing these measures could significantly reduce microbial contamination levels, thereby improving the safety and quality of pastries offered to consumers.

Our results revealed that *S. aureus* was the most abundant species identified, accounting for 21.43% of the isolates, followed by *M. luteus* and *E. coli* with 12.5% of the isolates of each. These results are consistent with previous studies that document its frequent presence in food production due to its resilience and adaptability (Arabestani et al., 2022; Kotzekidou, 2013; Necidová et al., 2022; Khakipour and Dastpeyman, 2021). Moreover, these findings highlight the diverse microbial ecology inherent to pastry shop environments and underscore the importance of comprehensive microbial surveillance and control measures to mitigate contamination risks effectively (Titouche et al., 2023; Da Silva et al., 2020).

AST results showed high resistance to erythromycin and tetracycline, with some isolates identified as MRSA and ESBL-producers. Notably, the identification of MRSA among a substantial proportion of *S. aureus* isolates, as well as the detection of ESBL-producing strains of *E. coli* and *K. pneumoniae*, highlights the prevalence of clinically significant antimicrobial resistance mechanisms in our study population. These findings underscore the importance of surveillance and infection control measures to prevent the spread of antimicrobial-resistant pathogens in the community (Arabestani et al., 2022; Mekhloufi et al., 2021; Kuenzli et al., 2014).

Overall, this study, along with similar research, underscores the significant issue of microbial contamination in pastry products. The presence of pathogenic bacteria and drug-resistant strains poses a substantial risk to public health, highlighting the need for regular monitoring and strict control measures in pastry shops. Given that microbial contamination in pastries appears to be a widespread issue, implementing standardized safety measures is essential to protect consumers (AWINO FO, 2015; Grudlewska-Buda et al., 2023). To address these concerns, we recommend targeted training programs for pastry shop managers and workers, focusing on internationally recognized food safety standards. These programs should cover the Codex Principles of Good Hygiene Practices (GHP) and Good Manufacturing Practices (GMP) to ensure high standards of cleanliness and safe food handling. Additionally, building a strong food safety culture within these establishments is crucial. Training on Hazard Analysis and Critical Control Points (HACCP) would help staff systematically identify and control potential contamination risks. Such initiatives would improve workers’ understanding of hygiene practices, proper food handling, and maintaining sanitary conditions throughout the production process. Adopting these standards could significantly reduce microbial contamination in pastries, contributing to safer food products and enhancing the overall quality of food safety in the region’s confectioneries.

## 5 Conclusion

In conclusion, our findings indicate a high prevalence of microbial contamination in pastry shops in Markazi province, Iran. The presence of drug-resistant isolates, such as MRSA and ESBL-producing strains, emphasizes the importance of regular monitoring and control measures to prevent foodborne infections. Pastry shops must adhere to good manufacturing practices and implement proper hygiene protocols to ensure the safety of their products. Further research and collaboration are warranted to develop standardized guidelines and protocols to ensure the safety of pastries and other food products.

## Data Availability

The original contributions presented in the study are publicly available. This data can be found here: NCBI database, PA1 *N. asiatica* (OP520910), isolate PA3 *N. asteroides* (PV077341), isolate PA17 *E.coli* (PV077340), isolate PA24 *K. pneumoniae* (PV077343), isolate PA5 *S. aureus* (PV077342), isolate PA27 *S. epidermidis* (PV077344), isolate PA40 *S. warneri* (KY411690), and isolate PA30 *E. faecium* (PV077345)]. All other relevant data are available in the manuscript in the form of figures and tables.
