# Corrigendum: Molecular surveillance and antimicrobial susceptibility profile of bacterial contamination in pastries of Iranian confectioneries: a public health concern

**DOI:** 10.3389/fmicb.2025.1573099

**Published:** 2025-03-17

**Authors:** Shiva Hosseini, Tahereh Motallebirad, Mohammad Reza Mohammadi, Mehdi Safarabadi, Zeynab Beheshti, Mohammad Ali Orouji, Omid Mardanshah, Davood Azadi

**Affiliations:** ^1^Department of Genetics, Marvdasht Branch, Islamic Azad University, Marvdasht, Iran; ^2^Department of Research and Development, Satras Biotechnology Company, Islamic Azad University of Khomein, Khomein, Iran; ^3^Department of Bacteriology, Faculty of Medical Sciences, Tarbiat Modares University, Tehran, Iran; ^4^Department of Nursing, Khomein University of Medical Sciences, Khomein, Iran; ^5^Department of Nursing, Khomein Branch, Islamic Azad University, Khomein, Iran; ^6^Department of Parasitology and Mycology, School of Medicine, Isfahan University of Medical Sciences, Isfahan, Iran; ^7^Department of Biology, Faculty of Basic Sciences, Lorestan University, Khorramabad, Iran

**Keywords:** pastries, microbial pollution, AST, 16s rRNA, microbial contamination

In the published article, there was an error. The accession numbers of isolate PA3 *N. asteroides* (OQ195247), isolate PA17 *E.coli* (OQ195249), isolate PA24 *K. pneumoniae* (OQ195250), isolate PA5 *S. aureus* (OQ195254), isolate PA27 *S. epidermidis* (OQ195256), and isolate PA30 *E. faecium* (OQ195258), were wrong.

A correction has been made to Section 3 Results, *3.1 16S rRNA gene sequence accession numbers*. This sentence previously stated:

“The GenBank accession numbers for the 16S rRNA gene sequences of isolated bacteria in this study are listed below, isolate PA1 *N. asiatica* (OP520910), isolate PA3 *N. asteroides* (OQ195247), isolate PA17 *E.coli* (OQ195249), isolate PA24 *K. pneumoniae* (OQ195250), isolate PA5 *S. aureus* (OQ195254), isolate PA27 *S. epidermidis* (OQ195256), isolate PA40 *S. warneri* (KY411690), and isolate PA30 *E. faecium* (OQ195258).”

The corrected sentence appears below:

“The GenBank accession numbers for the 16S rRNA gene sequences of isolated bacteria in this study are listed below, isolate PA1 *N. asiatica* (OP520910), isolate PA3 *N. asteroides* (PV077341), isolate PA17 *E.coli* (PV077340), isolate PA24 *K. pneumoniae* (PV077343), isolate PA5 *S. aureus* (PV077342), isolate PA27 *S. epidermidis* (PV077344), isolate PA40 *S. warneri* (KY411690), and isolate PA30 *E. faecium* (PV077345).”

A correction has been made to the Data Availability Statement. This sentence previously stated:

“[NCBI database, PA1 *N. asiatica* (OP520910), isolate PA3 *N. asteroides* (OQ195247), isolate PA17 *E.coli* (OQ195249), isolate PA24 *K. pneumoniae* (OQ195250), isolate PA5 *S. aureus* (OQ195254), isolate PA27 *S. epidermidis* (OQ195256), isolate PA40 *S. warneri* (KY411690), and isolate PA30 *E. faecium* (OQ195258)].”

The corrected sentence appears below:

“[NCBI database, PA1 *N. asiatica* (OP520910), isolate PA3 *N. asteroides* (PV077341), isolate PA17 *E.coli* (PV077340), isolate PA24 *K. pneumoniae* (PV077343), isolate PA5 *S. aureus* (PV077342), isolate PA27 *S. epidermidis* (PV077344), isolate PA40 *S. warneri* (KY411690), and isolate PA30 *E. faecium* (PV077345)].”

The authors apologize for this error and state that this does not change the scientific conclusions of the article in any way. The original article has been updated.

