# Correction: Molecular surveillance and antimicrobial susceptibility profile of bacterial contamination in pastries of Iranian confectioneries: a public health concern

**DOI:** 10.3389/fmicb.2026.1883665

**Published:** 2026-06-02

**Authors:** Shiva Hosseini, Tahereh Motallebirad, Mohammad Reza Mohammadi, Mehdi Safarabadi, Zeynab Beheshti, Mohammad Ali Orouji, Omid Mardanshah, Davood Azadi

**Affiliations:** 1 Department of Genetics, Marvdasht Branch, Islamic Azad University, Marvdasht, Iran; 2 Department of Research and Development, Satras Biotechnology Company, Islamic Azad University of Khomein, Khomein, Iran; 3 Department of Bacteriology, Faculty of Medical Sciences, Tarbiat Modares University, Tehran, Iran; 4 Department of Nursing, Khomein University of Medical Sciences, Khomein, Iran; 5 Department of Nursing, Khomein Branch, Islamic Azad University, Khomein, Iran; 6 Department of Parasitology and Mycology, School of Medicine, Isfahan University of Medical Sciences, Isfahan, Iran; 7 Department of Biology, Faculty of Basic Sciences, Lorestan University, Khorramabad, Iran

**Keywords:** pastries, microbial pollution, AST, 16s rRNA, microbial contamination

In the published article, an incorrect accession number was published. In **Materials and methods**, ***3.1. 16S rRNA gene sequence accession numbers*, page 5**, paragraph 1.

Within the 16SrRNA gene sequence accession numbers, the accession number of *S. warneri* strain was a wrong isolate; PA40 KY411690. The correct accession number is, isolate PA40 *S. warneri PZ413541*.

The incorrect sentence stated:

“The GenBank accession numbers for the 16S rRNA gene sequences of isolated bacteria in this study are listed below, isolate PA1 N. asiatica (OP520910), isolate PA3 N. asteroides (OQ195247), isolate PA17 *E. coli* (OQ195249), isolate PA24 *K. pneumoniae* (OQ195250), isolate PA5 *S. aureus* (OQ195254), isolate PA27 *S. epidermidis* (OQ195256), isolate PA40 *S. warneri* (KY411690), and isolate PA30 *E. faecium* (OQ195258).”

The corrected sentence is below:

“The GenBank accession numbers for the 16S rRNA gene sequences of isolated bacteria in this study are listed below, isolate PA1 *N. asiatica* (OP520910), isolate PA3 *N. asteroides* (PV077341), isolate PA17 *E. coli* (PV077340), isolate PA24 *K. pneumoniae* (PV077343), isolate PA5 *S. aureus* (PV077342), isolate PA27 *S. epidermidis* (PV077344), isolate PA40 *S. warneri* (PZ413541), and isolate PA30 *E. faecium* (PV077345).”

The original article has been updated.

